# The upside: coping and psychological resilience in Australian adolescents during the COVID-19 pandemic

**DOI:** 10.1186/s13034-021-00432-z

**Published:** 2021-12-18

**Authors:** Joanne R. Beames, Sophie H. Li, Jill M. Newby, Kate Maston, Helen Christensen, Aliza Werner-Seidler

**Affiliations:** Black Dog Institute, University of New South Wales, Sydney, NSW Australia

**Keywords:** Resilience, Coping, Adolescent, Youth mental health, Covid-19, Pandemic

## Abstract

**Background:**

Since the COVID-19 outbreak, few studies have investigated the positive psychological consequences on young people. This study examined resilience, positive experiences, and coping strategies reported by Australian adolescents during COVID-19.

**Methods:**

Self-report surveys were administered online to a sample of 760 Australian adolescents aged 12–18 years. Quantitative and qualitative methods were used to assess resilience, positive experiences, and coping strategies. Exploratory regression analyses were conducted to explore the relationship between resilience and demographics and mental illness history, as well as between resilience and positive experiences.

**Results:**

Overall, adolescents were somewhat resilient (*M* = 20.93, *SD* = 8.29). They reported positive experiences during COVID-19, including increased empathy, compassion, gratitude, and connection with others, and reported using a range of active coping strategies. Having a mental illness history and identification as female or non-binary gender were associated with lower resilience (*B*s > 2.82, *p*s < 0.001). Further, resilience was associated with decreased psychological distress (*OR* = 0.89, *p* < 0.001) and with increased positive experiences (*OR*s > 1.03, *ps* < 0.001).

**Conclusions:**

Our results indicate that Australian adolescents commonly reported positive experiences and used active coping strategies during COVID-19. Some young people demonstrated higher levels of resilience and were able to make the most out of an unpredictable situation that severely disrupted their daily routine. However, further prospective research using longitudinal methods is necessary to examine causal relationships between variables. An implication of our findings is that resilience-building programs for adolescents may be effective in increasing adaptability after adversity (e.g., climate change, bushfires, pandemics).

## Background

The COVID-19 pandemic has had a profound effect on adolescents around the world [1]. Young people have experienced disruptions to their education, social connections, family relationships, future job opportunities, financial stability, and mental health. These negative consequences have been documented in a growing number of cross-sectional and longitudinal surveys, with the overwhelming consensus being that the mental health of adolescents has deteriorated during the pandemic [1–3]. However, it is unknown whether adolescents have demonstrated psychological resilience, experienced any positive effects, or used effective coping strategies during COVID-19. Understanding how adolescents have coped will facilitate disaster planning by guiding ways to optimise social resources and enhance psychological resilience [4].

Psychological resilience refers to the maintenance or recovery of mental health after times of adversity [5–7]. According to the developmental systems perspective, adaptive capacity depends on multiple interacting systems e.g., [8–11]. For example, a young person is embedded in systems such as family and school, which are, in turn, embedded in higher order systems such as community and economies. The systems perspective hypothesises that the process of adaptation depends on the capacity of these systems to adapt in response to threat. The shift toward the systems perspective was largely influenced by the growing threat of mass-trauma global adversities, including terror attacks, natural environmental disasters, and pandemics [9, 12]. In the current paper, we focus on the individual-level. We answer questions about how young people coped during the COVID-19 outbreak and investigate factors or processes that support adaptive success [8]. Individual-level factors that promote resilience in adolescents include age and gender, as well as active coping strategies, hope, and optimism [7, 13]. Overall, individuals show varying levels of resilience in response to stressful life events [8].

The use of effective coping skills to regulate emotional experiences during, or after, adversity is an example of an adaptive process underpinning resilience [7]. Coping skills can be conceptualised in different ways [14], including differentiating between active (or approach) and passive (or avoidant) coping skills. Active coping involves using cognitive and behavioural strategies to directly reduce or control stress, such as problem-solving, seeking social support, and cognitive restructuring, whereas passive coping involves avoiding or disengaging from sources of stress [15]. In general, active coping strategies are related to better adjustment to stress and improved mental health compared to passive strategies [15, 16]. Similarly, research has also shown that active coping strategies, such as problem-solving and social support seeking, are important in the transition from adolescence to early adulthood [17]. Taken together, this research indicates that the use of active coping skills may be indicative of resilience in response to COVID-19.

Several international studies have examined youth resilience in the context of COVID-19. One cross-sectional survey study with Chinese youth conducted in April 2020 found that trait resilience and use of positive coping strategies were related to decreased depression and anxiety [18]. Positive coping encompassed active (rather than passive) strategies, including cognitive reappraisal, problem-solving, and help-seeking. Converging results have been found in the Unites States and Europe, with young people reportedly using social connection, relaxation, staying busy, hobbies, watching television or playing video games, and maintaining a routine as ways to cope [19–22]. Another survey study with young adults (*M*_age_ = 22) in Zurich, similarly identified that coping strategies, such as keeping a daily routine, physical activity, contacting friends and family, acceptance of the COVID-19 crisis, and cognitive restructuring, were associated with reduced distress during the pandemic [23]. The results of these studies show the beneficial effects of resilience processes on youth mental health during COVID-19. It remains unknown whether similar benefits were experienced by young Australians, during a later phase of the pandemic that included stringent lockdowns.

The relevance of resilience processes in the context of COVID-19 is underscored by an emerging adult literature. Global studies show that engagement in active coping strategies, such as recreational activity, acceptance, and perspective taking, were associated with lower symptoms of depression, anxiety, and stress during COVID-19 [24, 25]. Results from a New Zealand adult sample also show pandemic ‘silver linings’ including a sense of community and social cohesion, improved social relationships, personal reflection, personal development, and perceived agency [26]. Together, these results indicate that the effect of COVID-19 may not be inherently or exclusively negative and that some individuals may be more likely to experience positive outcomes than others. Further investigation is needed to determine whether these findings generalise to adolescents, a group that typically undergoes a unique set of developmental changes and major life transitions.

## The current study

The potential positive aspects of young people’s experience during COVID-19 in the Australian context is unknown. Further, limited attention has been given to individual differences in resilience and the relationship between resilience and positive and negative experiences during the pandemic. We addressed these gaps by conducting a large cross-sectional, mixed methods survey study to investigate resilience, positive experiences, and coping strategies in Australian adolescents during COVID-19. In line with process-oriented resilience frameworks [7], including the developmental systems perspective [8], resilience was conceptualised as a dynamic process of adaptation.

The aims of this mixed-methods study were two-fold. The first aim was to explore resilience, positive experiences, and active coping strategies in Australian adolescents between 12 and 18 years. For the qualitative component, young people were asked to answer open-ended questions about coping strategies employed during the pandemic. Based on previous studies from the youth and adult literature during COVID-19 [18, 19, 21, 24], we expected that adolescents would report positive experiences and engagement in primarily active (versus passive) coping strategies. The second aim was to investigate associations between resilience and demographic characteristics (age, gender, mental illness history), distress, and positive experiences. Given the early stage of COVID-19 research in youth sample, our analysis of the associations between resilience and other variables were exploratory.

## Method

### Participants

Young people between the ages of 12–18 years were recruited across Australia via social media advertisements. Data was collected from an online survey between the end of June 2020 and the beginning of August 2020. This period of data collection included the relaxing of lockdown restrictions across Australia, with the exception of the city of Melbourne and the state of Victoria (both were subject to restrictive second wave lockdown conditions during this time). Data reported here were collected as part of a larger survey examining the impact of COVID-19 on the lives and mental health of Australian adolescents, which is documented elsewhere [27].

### Measures

#### Demographics and mental illness history

Information was collected on participants’ age and gender. For mental illness history, participants were asked whether they had ever been diagnosed with depression or anxiety by a professional (0 = no, 1 = yes, depression only, 2 = yes, anxiety only, 3 = yes, both depression and anxiety, 4 = I don’t know, 5 = prefer not to say).

#### Resilience

The 10-item Connor-Davidson Resilience Scale (CD-RISC-10) was used to measure resilience [28]. The CD-RISC-10 assesses the availability of resilience factors, such as social support and self-efficacy, to maintain or regain mental health despite adversity. Each item is rated on a five-point scale ranging from 0 = not true at all, 1 = rarely true, 2 = sometimes true, 3 = often true, and 4 = true nearly all of the time. Total scores are calculated by summing all items, with a higher score indicating higher resilience. Scores range from 0 to 40. The CD-RISC-10 has demonstrated reliability and validity among adolescent samples [29, 30]. There are no defined cut-off scores for the scale, however normative data indicated that the mean resilience score in an international adolescent sample was 24.7 [31]. To the best of our knowledge, normative data for the CD-RISC-10 for Australian adolescents are not yet available.

#### Psychological distress

The Kessler-6 (K6) assessed general psychological distress over the past 30 days [32, 33]. Each item is rated on a 5-point scale ranging from 1 = none of the time, 2 = a little of the time, 3 = some of the time, 4 = most of the time, 5 = all of the time. Total scores are calculated by summing all items, with a higher score indicating higher psychological distress. Scores range from 6–30. Consistent with recommended cut-off scores, responses were then binarised into no probable mental illness (scores 6–18) and probable mental illness (19–30; [34]). The K6 has been widely used and validated with young people and has strong psychometric properties [35–38]. Overall, the K6 is appropriate to measure adolescent distress in large surveys.

#### Positive experiences

A bespoke questionnaire developed by the authors for the current study was used to assess positive experiences during COVID-19, based on previous literature [39, 40]. Participants were presented with a list of five positive experiences, including: “feeling more connected with friends and family”, “feeling things are more calm at home”, “feeling more grateful”, “feeling kinder and more generous towards others”, and “feeling more empathy towards others who are less fortunate than you”. Participants were asked to indicate whether they had experienced any of these outcomes in the past week by selecting a check box.

#### Coping strategies

Participants were asked to write free text responses to one open-ended question that enquired about coping strategies used during COVID-19 (i.e., “What strategies have you used to cope?”). Responses were optional and not required to complete the survey.

### Procedure

Participants were directed to the online survey platform (Qualtrics) after clicking on study advertisements. All respondents were required to demonstrate that they understood the study and had the capacity to provide informed consent, using the Gillick Competency Task [41], before providing consent and accessing the survey. Upon completion of the survey, participants were placed into a draw to receive one of five AUD$50 vouchers. The study was approved by the UNSW Human Research Ethics Committee (HC200334).

## Statistical analysis

### Quantitative analyses

All quantitative analyses were conducted in SPSS v. 25. Descriptive analyses were used to report demographic and sample characteristics, and the proportion of young people who endorsed each positive experience during the pandemic.

A simultaneous multiple regression analysis was conducted to explore the relationship between demographic characteristics and resilience. Resilience was regressed on age, gender, and mental illness history (i.e., a previous diagnosis of depression and/or anxiety). Gender and mental illness history were entered into the model using dummy codes (0 = male, 1 = female; 2 = non-binary/other; 0 = no diagnosis; 1 = diagnosis). Unstandardised regression coefficients were used to describe the effects. Squared semi-partial correlations were examined to further understand the unique relationships between the variables within the model.

A binary logistic regression analysis was conducted to explore the relationship between resilience and psychological distress (0 = no probable mental illness, 1 = probable mental illness). For this analysis, psychological distress was regressed on resilience. A series of binary logistic regression analyses were then conducted to explore the relationship between resilience and each positive experience (e.g., increased empathy; 0 = No, 1 = Yes). For these analyses, positive experiences were regressed on resilience. Unstandardised estimates were exponentiated into odds ratios. All regression assumptions, including normality, homoscedasticity, linearity, and multicollinearity were met within the relevant models. Excluding outliers in the regression analyses did not change the pattern of results, and so all outliers are included in the results below.

### Qualitative analyses

#### Coding strategy

All qualitative analyses were conducted in Excel. One author (JRB) coded responses to the open-ended question using a deductive approach. JRB developed a code book based on coping strategies typically defined as “active” and “passive” in the coping literature [42], as well as coping strategies identified in previous COVID-19 research [18, 23]. JRB refined the codes through repeated immersion with the responses. In line with the study aim, focus was given to positive or helpful strategies rather than those that were clearly maladaptive or harmful, such as self-harm. Passive strategies were coded when they were described as alleviating emotional distress or promoting positive emotions, even if only in the short term. This approach aligns with emerging research that indicates that coping or emotion regulation strategies are not inherently maladaptive or adaptive [43]. A second author (SL) applied the code book to 20% of the responses to check coding alignment. There was high agreement between both coders (90%) and all discrepancies were resolved through discussion.

## Results

### Sample characteristics

The final sample included 760 young people aged between 12 and 18 years, with a mean age of 14.8 years (*SD* = 1.26). A majority were female (72%; 5% non-binary), spoke English at home (87.7%) and born in Australia (88.1%), and 9.4% identified as Aboriginal or Torres Strait Islander. Participants lived across all Australian states and territories, with the majority located in Victoria (*n* = 266, 35.2%), New South Wales (*n* = 238, 31.5%) and Queensland (*n* = 116, 15.3%). There were no significant differences between location and resilience, *F*(7, 739) = 0.16, *p* = 0.99, and so data were not segregated by location for the current analyses. Approximately 50% of the sample indicated that their parent or carer’s job had been impacted by the pandemic. Just over 35% reported being diagnosed with anxiety and/or depression in the past. The mean score on the K6 was 18.08 (*SD* = 6.63; range 6–30). Almost half of the sample (48.5%) scored above the threshold that indicates psychological distress indicative of probable mental illness. For more detailed characteristics of the sample, see [27].

### Analyses

#### Resilience

The mean level of resilience reported by young people was 20.93 (*SD* = 8.29, range = 0–40).

#### Individual differences in resilience

A simultaneous multiple linear regression was run to explore the relationship between resilience and age, gender, and mental illness history (i.e., a previous diagnosis of depression and/or anxiety). The overall model was significant, *F*(4, 656) = 23.31, *p* < 0.001, *R*^*2*^ = 12.4%. Female gender, non-binary gender, and mental illness history explained a significant proportion of the variance in resilience (*p*s < 0.001), whereas age did not (*p* = 0.31). Young people who identified as female and young people who identified as non-binary or another gender reported significantly lower resilience levels than young people who identified as male. Young people with a mental illness history reported significantly lower resilience compared to those without a mental illness history. Squared semi-partial correlations indicated that mental illness history (*sr*^*2*^ = 0.067; 6.76%) accounted for a greater unique proportion of the variance in resilience compared to age (*sr*^*2*^ = 0.0014; 0.14%) and the gender variables (*sr*^*2*^ < 0.022; < 2.2%). See Table 1 for regression model output.

**Table 1:** Linear regression results for resilience

	*B*	*95% CI for B*		*SE B*	*β*	*t*	*p*	*R*^*2*^
		*LL*	*UL*					
Model								0.12
Constant	29.31	21.94	36.68	3.75		7.81	0.00	
Age	− 0.25	− 0.74	0.23	0.25	− 0.04	− 1.02	0.31	
Female	− 2.83	− 4.39	− 1.27	0.79	− 0.15	− 3.57	0.00*	
Non-binary	− 0.35	− 7.94	− 2.75	1.32	− 0.17	− 4.05	0.00*	
Mental illness history	− 4.61	− 5.88	− 3.34	0.65	− 0.27	− 7.13	0.00*	

*B* unstandardised regression coefficient, *CI* confidence interval, *LL* lower limit, *UL* upper limit, *SE B* standard error for the unstandardised coefficient, *β* standardised regression coefficient, *R*^*2*^ coefficient of determination

^*^p < 0.001

#### Resilience and psychological distress

A binary logistic regression was run to explore the relationship between resilience and psychological distress. The overall model was significant, χ^2^(1) = 134.92, *p* < 0.001, and explained 22% (Nagelkerke *R*^*2*^) of the variance in distress. Young people who reported higher resilience had lower odds of reporting probable mental illness compared to young people who reported higher resilience (*OR* = 0.89, *p* < 0.001). See Table 2 for regression model output.

**Table 2:** Logistic regression for psychological distress

	*OR*	*95% CI for OR*		*p*
		*LL*	*UL*	
Resilience	0.89	0.87	0.91	0.00*
Constant	11.03			0.00

*OR* odds ratio, *CI* confidence interval, *LL* lower limit, *UL* upper limit

^*^p < 0.001

#### Positive experiences

Over half of the sample (56.9%) reported feeling greater levels of empathy toward others who are less fortunate than themselves, and 42.9% reported feeling more grateful in general. Approximately one-third of the sample reported feeling more connected with friends and family (34%) and feeling kinder and more generous toward others (32.1%). In comparison to these positive experiences, experiencing feelings that things were calmer at home was endorsed by fewer young people (23%). Further, the logistic regression models indicated that resilience was significantly related each type of positive experience, *OR*s > 1.03, *ps* < 0.001. For example, young people with higher resilience also had higher odds of feeling more connected to friends and family compared to those who did not report feeling more connected. The amount of variance explained by each positive experience was low and variable, ranging from 2.3 to 9.5%. See Table 3 for the regression model output.

**Table 3:** Logistic regressions for positive experiences

	*OR*	*95% CI for OR*		*p*
		*LL*	*UL*	
Feeling connected				
Resilience	1.06	1.04	1.08	0.00*
Constant	0.14			0.00
Calm at home				
Resilience	1.04	1.02	1.08	0.00*
Constant	0.12			0.00
Feeling grateful				
Resilience	1.07	1.05	1.09	0.00*
Constant	0.17			0.00
Feeling kind/generous				
Resilience	1.05	1.03	1.07	0.00*
Constant	0.18			0.00
Feeling empathy				
Resilience	1.03	1.01	1.05	0.00*
Constant	0.69			0.07

*OR* odds ratio, *CI* confidence interval, *LL* lower limit, *UL* upper limit

^*^p < 0.001

#### Coping strategies

The coding of free-text responses to the question assessing coping strategies resulted in 16 categories. Of these categories, 14 were of active coping strategies and 2 were of passive coping strategies. See Table 4 for a summary of descriptive statistics for all categories reported and Table 5 for definitions and example quotes.

**Table 4:** Summary of descriptive statistics for coded coping strategies

Coping strategy	*n*	%
Active		
Socialising	180	37.89
Hobbies	116	24.42
Physical exercise	60	12.63
Psychological strategies	42	8.84
Routine	34	7.16
Focusing on the positives	31	6.53
Help-seeking	27	5.68
Emotional expression	21	4.42
Psychological treatment/therapy	21	4.42
Time for self	11	2.32
Limit screen time	6	1.26
Spirituality/religion	4	0.84
Being informed	3	0.63
Humour	2	0.42
Passive		
Distraction	51	10.74
Rest/sleep/relax	29	6.11

**Table 5:** Summary of categories in open-ended responses about coping strategies used during COVID-19

Coping strategy	Definition	Example
Active		
Socialising	Talking, hanging out, or connecting with family or friends (face-to-face or using technology)	“Talking to friends and family” “I video call my friends in some of my classes and after school so I'm not lonely. We often do schoolwork, homework, exercise/workouts or just hang out”
Hobbies	Any kind of activity enacted for enjoyment, pleasure, or achievement, such as listening to music, art, reading, or gaming	“Doing what i like, my hobbies. drawing, dancing, music, eating” “Indulging myself in activities I enjoy, like music and gaming”
Physical exercise	Any kind of physical activity, such as team sports, walking, running, yoga, or other references to working out	“Going outside and exercising” “Walking a few hours a day”
Psychological strategies	Active coping strategies, such as breathing, perspective taking, using calm/relaxation apps, and meditation	“I have used breathing exercises to calm myself down” “I tried most of the anxiety strategies I leant [sic] when I saw a psychologist last year (eg. Socratic questions, meditation, staying in the moment, etc.)”
Routine	Deliberate engagement in regular activities, such as schoolwork/study (or returning to school), making plans and to-do lists, basic hygiene (e.g., brushing teeth, getting dressed), or daily chores (e.g., making bed), eating healthily	Having a schedule especially with school work, arranging them into hours of the day and giving myself breaks in between
Focusing on the positives	Taking a positive outlook on the current situation and the future, or other references to doing the best that they can	“I try to stay optimistic” “Just saying it will be okay, you can make it. This will be better soon”
Help-seeking	Asking for help from friends, family, teachers, or professionals, including broad references to talking to other people about problems	“I've been talking to my parents more (about mental health) and I've found that this helps” “I spoke to a teacher that I have for multiple classes ab[out] a couple of the things I’ve been struggling with after I had a incident in her class”
Emotional expression	Outward displays of emotions to self or to others (e.g., crying), including through drawing/writing	“Honestly I’ve just cried a lot” “I have a sketch book where I draw my emotions and thoughts”
Psychological treatment/therapy	Seeing a mental health professional for support and/or engaging in therapy (including counselling, psychologists, medications)	“I’ve been seeing a psychologist” “Talking to my therapist”
Time for self	Taking time out to reset and spending time on own (but not explicitly framed as a way to avoid problems)	“Spending time to concentrate on a particular task, and chilling for my own self”
Limit screen time	Restricting the amount of time spent using technology	“I have set myself up a screen time limit to reduce my screen time”
Spirituality/religion	Any reference to religion, spirituality, or God	“Have faith in God” “Reading my bible”
Being informed	Staying up to date with information about COVID-19	“Watching the news so I knew exactly what was going on and no one else was telling me false stuff”
Humour	Any reference to humour	“Humour”
Passive		
Distraction	Deliberately not paying attention or trying to distract from the current situation, or other references to keeping busy (includes TV/Netflix/YouTube)	“Focusing on something away from anything involving the virus” “trying to keep my mind off the pandemic and issues going on at the moment”
Rest/sleep/relax	Any mention of resting, sleeping, napping, or relaxing (coded as separate to routine when identified as a coping strategy to boost functioning, rather than maintaining regular sleep/wake cycles)	“Mainly just having a good nights’ sleep”

A total of 596 (78.42%) young people provided a response to the open-ended coping strategies question. Of these, 475 (79.70%) responses were coded as coping strategies that had the potential for positive effects on mental health and wellbeing. The total number of coping strategies used ranged between 1 and 4, with a mean of 1.40 (*SD* = 0.67). The most common coping strategies were active, including socialising (37.89%), engaging in hobbies (24.4%), and doing physical exercise (12.63%). Other active coping strategies reported included using psychological strategies such as perspective taking and relaxation (8.84%), keeping a routine (7.16%), focusing on the positives (6.53%), and help-seeking (5.68%). Overall, passive coping strategies, including distraction (10.74%) and sleeping or relaxing/sleeping (6.11%), were less frequently reported than active coping strategies.

## Discussion

To the best of our knowledge, this study is the first to explore the positive aspects of young people’s psychological experience during COVID-19 within the Australian context. Our results add to the emerging literature showing that the experience of young people during COVID-19 is not exclusively negative, and that positive experiences are common.

Resilience levels in our sample were slightly lower than normative levels reported in an international sample with a similar age range before COVID-19 [31]. Resilience levels were also lower than a large representative Australian study of older adolescents between 16–17 years (i.e., 26.5/40 on the CD-RISC-10; [44]). Increased psychological distress has been demonstrated in young people during COVID-19 e.g., [2, 27]. Given our finding that there is a negative relationship between resilience and distress, it is not surprising that resilience levels in our sample of young people were comparatively reduced overall during the pandemic. Young people are vulnerable to stressors relating to family, friends, and schools [45], all of which have been significantly disrupted by COVID-19. Our results also indicated that some young people were less resilient than others (e.g., those who identified as female or non-binary, those who reported a history of anxiety/depression). Identifying those with increased vulnerability will be important to aid optimal recovery in response to future stressors (e.g., climate change, pandemics, bushfires). Resilience-building programs are one way to facilitate adaptive functioning in the face of adversity. Resilience-building interventions generally aim to strengthen protective factors [6], which can be internal (e.g., coping skills) or external (e.g., family relationships, community support and available services). Many resilience-based approaches are now available, with increasing evidence that they help to prevent decline or regain psychological functioning in adolescence after adversity [46–48]. Further intervention research is necessary to determine the beneficial effects of these approaches for specific sub-groups of young people (e.g., different ages, genders, sexualities).

In our sample of young people, mental illness history was more strongly related to resilience compared to age and gender. This finding corresponds to prior COVID-19 research in young people showing that mental illness history is related to higher distress [18, 27]. Young people with a mental illness history may be more vulnerable to increased threat caused by COVID-19. Vulnerability may be explained by having fewer skills to manage distress and facilitate adaptation, or by an inability to use acquired coping skills effectively when the broader system is threatened. This interpretation aligns with research showing that effective emotion regulation is dependent on context [49], with flexible selection and timing of strategy use linked to better emotional outcomes e.g., [43, 50].

One resilience-building skill that might facilitate bouncing back from adversity is active coping. Evidence for the use and effectiveness of active coping strategies during COVID-19 has been documented in previous research [18, 19, 23], which we replicated in our cross-sectional study. We found that the most reported strategies were socialising, engaging in hobbies, and exercise. These findings emphasise the importance of peer relationships for young people [51], as well as their capacity to engage in activities that potentially enhance positive mood. We also found evidence for the use of passive coping strategies, including distraction and sleep or relaxing. Although our cross-sectional findings cannot extrapolate causality, based on the resilience framework [7], the use of coping strategies likely increases the likelihood of positive adaptation.

In addition to coping strategies, other important psychosocial resilience factors include optimism and hope [52]. Adolescents in our sample reported feeling more empathy, gratitude, connectedness, and kinder, more generous feelings toward others. Increased resilience was associated with these positive experiences, converging with other research that has linked challenging experiences with wellbeing and reduced psychopathology [53]. These results are also consistent with findings that adults have the capacity to experience ‘silver linings’ and unexpected positive outcomes in the midst of the COVID-19 pandemic [26]. Future research is needed to determine whether positive experiences can be sustained, and whether adolescents with an affinity for focusing on the positives are more resilient than those who do not (or vice versa).

Our exploratory results indicate that gender might influence vulnerability to adversity and, by extension, capacity for positive experiences. Young people who identified as female or non-binary/another gender reported significantly lower resilience. Although a comparison to pre-pandemic levels was not available, this general pattern of results is consistent with prior research [18, 54, 55]. Given that young females have higher rates of mental health problems, particularly internalising disorders such as anxiety and depression, they may be more vulnerable during prolonged stressors. Gender differences in resilience are not well understood and further research is needed to explore how gender affects vulnerability to and recovery after stressful life events. Identifying vulnerable individuals who are struggling to cope will help allocate mental health resources to those who need them most in the aftermath of COVID-19.

## Limitations

The current study had several limitations. Given our focus on internalising symptoms (e.g., psychological distress), we did not examine externalising disorders. Examining the relationship between resilience and externalising disorders in the context of large-scale public health emergencies is an area for future research. We also used a convenience sample of young people that was recruited online using established networks within the Black Dog Institute. Although this approach facilitated timely administration and data collection, which was necessary in the context of a rapidly evolving public health disaster, selection bias may limit generalisability to the broader population of Australian young people. For example, 72% of our sample were female, 9.4% identified as Aboriginal and/or Torres Strait Islander, and 88.1% were born in Australia. These percentages are higher than recent population estimates of gender, First Nations identification, and country of origin among Australian young people [56]. Sampling methods have been identified as a critical issue in COVID-19 research [57].

Another limitation was that our data collection methods relied on retrospective self-reports and open-ended responses from young people. This methodology is susceptible to response bias, such as social desirability, which can occur even when surveys are anonymous. Integration of other perspectives, such as from parents, school teachers, or school counsellors/psychologists, and use of validated measures (e.g., of resilience, coping strategies or positive experiences), might help to corroborate reports from young people and increase reliability of findings in future studies. Subjective measures of resilience have received criticism in the literature. There are no “gold standard” measures of resilience, with all requiring additional validation work [58]. Specific to the CD-RISC-10, lack of normative data in Australian adolescents means that findings in the current sample are difficult to contextualise. However, the CD-RISC-10 is one of the most frequently used scales and has excellent psychometric properties [59]. Overall, our findings add to the extant literature on youth resilience and build the case for positive experiences in the context of COVID-19.

Finally, this study was cross-sectional and correlational. This design means that causal conclusions cannot be made about resilience, coping, and other positive outcomes, nor about the long-term positive effects of COVID-19 on Australian youth. We cannot compare the resilience levels reported in our sample to pre-pandemic levels. Repeated assessments of resilience over the long-term are important to explore changes in resilience levels as COVID-19 progresses (e.g., emergence of the Delta or Omicron variants) and in response to government and community initiatives. In particular, there is a pressing need for prospective longitudinal resilience studies [7] that assess multiple developmental systems (e.g., individual, family, and economic; 13). Assessing multiple levels has the advantage of documenting cascading consequences, whether positive or negative, of large-scale environmental stressors such as COVID-19. Understanding the full impact of COVID-19 is critical to developing effective mental health disaster readiness and response plans for young people.

## Conclusions

Our study showed that, during the COVID-19 pandemic, young Australians have demonstrated resilience, albeit some more than others, as well as the capacity for positive experiences. Our study also showed that a large proportion of young people reported using active coping strategies during the rapidly evolving, unpredictable circumstances that they found themselves in. Building on prior work, our results indicate that resilience and distress are important targets for youth psychological intervention in public health emergencies such as pandemics. A major question for public health authorities is how to improve and prepare young people for a response to ongoing pandemics, as well as future pandemics, disasters and other impending crises driven by climate change. Clearly a psychological disaster plan is needed. Drawing upon the strengths of young people and incorporating capacity building before disaster strikes is likely to increase resilient responding. A systems-level approach that helps young people to focus on the positives and to build a repertoire of coping strategies is needed to maximise beneficial outcomes in the long-term following pandemics.

## Data Availability

Not publicly available due to the sensitive nature of the data and ethical guidelines.
